# Evaluation and use of surveillance system data toward the identification of high-risk areas for potential cholera vaccination: a case study from Niger

**DOI:** 10.1186/1756-0500-5-231

**Published:** 2012-05-14

**Authors:** Jose Guerra, Bachir Mayana, Ali Djibo, Mahamane L Manzo, Augusto E Llosa, Rebecca F Grais

**Affiliations:** 1 Epicentre - 8, rue St-Sabin, 75011, Paris, France; 2 Direction régionale de la Santé Publique, Maradi, Niger; 3 Faculté des sciences de la santé, Université de Niamey, Niamey, Niger

## Abstract

**Background:**

In 2008, Africa accounted for 94% of the cholera cases reported worldwide. Although the World Health Organization currently recommends the oral cholera vaccine in endemic areas for high-risk populations, its use in Sub-Saharan Africa has been limited. Here, we provide the principal results of an evaluation of the cholera surveillance system in the region of Maradi in Niger and an analysis of its data towards identifying high-risk areas for cholera.

**Results:**

We evaluated the cholera surveillance data using a standard CDC protocol, through interviews with heads of the system, and a review of cholera data collected between 2006–2009. The surveillance system was found to be sufficiently reliable to be able to utilize the data for the detection of high risk areas for cholera vaccination. Temporal, geographic and socio-demographic analyses of cholera cases indicated that between 2006 and 2009, 433 cholera cases were reported in the Maradi region of Niger. Two deprived neighborhoods of the region’s capital city, Bagalam and Yandaka, represented 1% of the regional population and 21% of the cholera cases, reaching a yearly incidence rate of 3 per 1000 in 2006 and 2008, respectively.

**Conclusions:**

The results of this evaluation suggest that the reporting sensitivity of the surveillance system is sufficient, to appropriately classify the region as cholera endemic. Additionally, two overcrowded neighborhoods in the regional capital met WHO criteria for consideration for cholera vaccination.

## Background

In 2008, Africa accounted for 94% of the cholera cases reported to the World Health Organization (WHO). Niger reported a small fraction of these cases, although certain areas of the country face repeated epidemics [1,2]. From 2000 to 2008, Niger reported cholera outbreaks every year, mainly in the south of the country and totaling close to 6000 cases [2]. The region of Maradi has the highest population density in the country and regularly reports cholera cases [2].

Two safe and effective oral cholera vaccines (Dukoral and Shanchol) are now available and prequalified by WHO [3-5], with some evidence of induced herd immunity [6,7]. To optimize implementation in cholera-endemic areas, WHO guidance recommends targeting oral cholera vaccination to areas where culture-confirmed cholera has been detected in at least 3 of the past 5 years; and incidence rates are at least 1/1000 population in any of these years or high-risk areas or groups have been identified using information collected from local public health officials [8]. Thus, epidemiological knowledge of the burden of cholera in a specific area is required before cholera vaccination is recommended. Although WHO recommends the use of these vaccines in endemic areas, their use in Sub-Saharan Africa has been limited [9].

National cholera surveillance systems are an affordable way to collect epidemiological data before a cholera vaccination campaign is implemented. They must, however, be evaluated [10], and if sound, surveillance data can then be analyzed to identify high-risk areas suitable for cholera vaccination.

Here, we provide the results of an evaluation of the surveillance system in the region of Maradi, Niger and an analysis of its data towards identifying high-risk areas that may be considered for potential cholera vaccination.

## Methods

**Figure 1:**
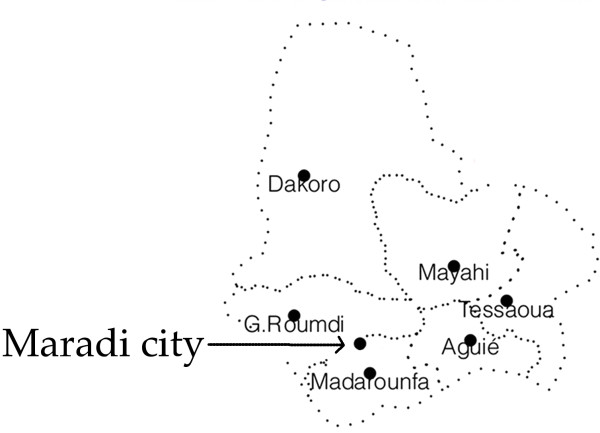
Map of the region of Maradi.

The region of Maradi is divided into 6 administrative districts (Figure 1). Maradi city is the regional capital and the second largest city in Niger. In collaboration with the regional health authorities, we first evaluated the surveillance system in the region following a standard protocol developed by the Centers for Disease Control and Prevention (Atlanta, Georgia, USA) [10]. Subsequently we analyzed this surveillance data, using WHO criteria to identify high-risk areas to target oral cholera vaccination in cholera endemic areas [8].

Briefly, the surveillance system evaluation covered the simplicity, flexibility, reactivity, stability, representativeness and acceptability of the system as well as the quality of data, the positive predictive value (PPV) and sensitivity. The evaluation, conducted in 2009 examines historical data since 2006 extracted from the Ministry of Health (MoH) records. It included reviewing all reports issued by the cholera surveillance system for the same period, including case reports about the onset of epidemics and response to epidemics. The evaluation consisted principally of detailed structured interviews with the heads of the cholera surveillance system of the Maradi region and with the heads of the 6 health facilities of the city of Maradi.

Using MoH surveillance records extracted for 2006–2009 and official population figures from the 2001 census, we estimated the cumulative incidence rates of cholera by year, administrative district and by neighborhood in Maradi city. For temporal analyses, we considered consultation date of the cholera cases and for geographic analyses the place of residence of cases. Available socio-demographic data (age and sex) were used to describe the cholera case population. For the identification of cholera endemic areas we apply the WHO definition of “the occurrence of fecal culture-confirmed cholera diarrhea in a population in at least 3 of the past 5 years” [8]. For the identification of high risk areas for potential cholera vaccination we applied WHO criteria of areas where 2 of the 3 following criteria are met: “(i) culture-confirmed cholera has been detected in at least 3 of the past 5 years; (ii) an incidence rate of cholera of at least 1/1000 population in any of these years has been recorded; (iii) if population-based incidence rates are not available, high-risk areas or groups have been identified using information collected from local public health officials” [8]. R statistical software [11] was used to analyze and present data collected from the surveillance system.

Participating heads of surveillance system orally consented to be interviewed. Authorization was provided by the Ministry of Health of Niger. No additional ethical committee approval was sought as we used routine surveillance data provided by the MoH. No supplementary interventions were conducted nor additional data collected for the evaluation and analysis presented here. All data were anonymous and neither ethnic nor identifying information was included.

## Results

The following paragraphs present first, an overview of the evaluation of the surveillance system, and second, an epidemiologic description of the surveillance system data to identify high risk-areas.

The surveillance system in Niger is based on WHO reference guidelines [12], covering 7 potential epidemic diseases, including cholera. No other surveillance system for cholera exists nationally. The surveillance system used the WHO standard case definition of cholera [12]:

During an inter-epidemic period: any individual aged 5 years old or more who presented acute dehydration or who died of acute watery diarrhea.

During an epidemic period: any individual aged 5 years old or more who presented an acute watery diarrhea with or without vomiting.

Both public and private health facilities are required to report cholera cases and related deaths for persons over 5 years of age. Health facilities report disease data to their sanitary districts (with one epidemiologist in charge). Each district reports its data to the sanitary region, which in turn reports to the MOH. During a cholera epidemic, especially at its onset, the heads of the surveillance system at the district and regional level conduct field investigations in the affected areas in order to identify related cases. A case of cholera is confirmed when either *Vibrio cholerae* O1 or O139 is isolated from the stool sample of a suspected case. A cholera epidemic is declared as soon as the first case of cholera is confirmed. Key attributes of the cholera surveillance system for the evaluation of data are presented in Tables 1 and 2. Complete results of the surveillance system evaluation are available elsewhere [13]. The evaluation of the cholera surveillance system of the Maradi region showed a good sensitivity of the system to detect the cholera cases and good quality of reported information for persons 5 years of age and older.

**Table 1:** Results of the interviews and data collection related with the data quality and sensitivity attributes of the surveillance system

**Attributes**	**Results of the interviews and data collection**
Data quality	
Completeness and validity of the data recorded	Very few missing values were found: 0/403 for the administrative district, 3/403 for age, 1/403 for sex. Only data from 2006 to 2009 were available at the regional level.
Sensitivity	
Sensitivity to detect cholera cases during an inter-epidemic period	Cholera cases were identified by the nurses in the healthcare facilities. Difficulties were reported in the detection of the first cases of cholera. Usually, the observation of a few cholera cases was necessary before reporting of suspected cases of cholera started.
Sensitivity to detect cholera cases during an epidemic period	During an epidemic, all the suspected cholera cases were referred to the cholera treatment centres (created at the onset of the epidemic), where they were documented. Surveillance system leaders conducted investigations to detect related cases and to inform the population about cholera. Radio messages were broadcasted to inform the population about cholera symptoms and encourage the reporting of any suspected case to the heads of healthcare facilities. Four heads of health structures reported that during epidemics, there was general awareness of the gravity of cholera and the population was more likely to seek care at healthcare facilities in case of acute diarrhea.
Sensitivity to detect a cholera epidemic	Each cluster of cholera cases was reported to the heads of the surveillance system. All interviewees felt confident that each cluster of cholera of cases was reported to the head of the surveillance system.

**Table 2:** Results of the interviews and data collection related with the positive predictive value and representativeness attributes of the surveillance system

**Attributes**	**Results of the interviews and data collection**
Positive predictive value	
Positive predictive value during an inter-epidemic period	For the initial fifth through tenth suspected cholera cases, stool samples were collected to perform stool cultures. This was done in the national laboratory in Niamey. Stool collection usually lacked for the first cholera cases. Tubes with Cary-Blair medium for the transport of the samples were not available from 2006 to 2009. For 26 cholera cultures results from 2006 to 2009 available at the time of the study, 13 were positive for *Vibrio cholerea* O1 ogawa (2 were positive for shigella and no pathogens were found for 11 cultures).
Positive predictive value during an epidemic period	Each epidemic was confirmed by stool culture. If an epidemic was confirmed with five to ten stool samples, stool collection and testing ceased. Four heads of health structures reported that during an epidemic, the population was aware of the gravity of cholera and that they were more likely to seek care at healthcare facilities in case of acute diarrhea.
Representativeness	
Accurate description of cholera cases over time	Each week, each healthcare facility completed the notifiable diseases reports, which include the number of cholera cases. When a cholera case is suspected, the head of the healthcare facility promptly contacted the district epidemiologist, who in turn contacted the regional responsible of the surveillance system. During an epidemic, the count of cholera cases was reported daily to the regional and national heads of the surveillance system.
Accurate description of cholera cases by geographic location	In remote villages, the first cholera cases did not seek care at healthcare facilities. After a few cases had been noted, the population tended to seek care at health facilities.
Accurate description of cholera cases by socio-demographics characteristics	For each cholera case, specific forms were completed with accurate information including: age, sex, address, consultation date, report date, date of symptom onset, vital status, and final diagnosis.Individuals aged less than 5 years are not typically reported. Some cases in children under 5 were reported, however.

**Figure 2:**
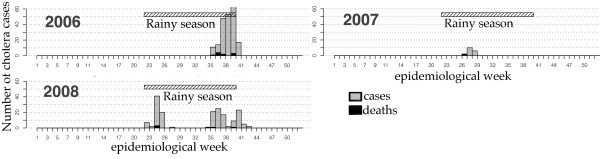
Temporal distribution of cholera cases in the region of Maradi.

**Figure 3:**
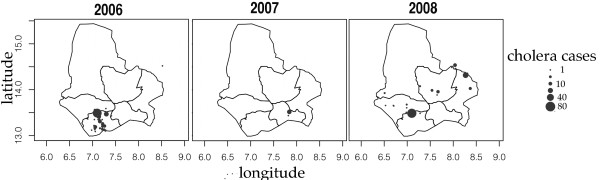
Distribution of cholera cases in the region of Maradi over 3 years.

**Figure 4:**
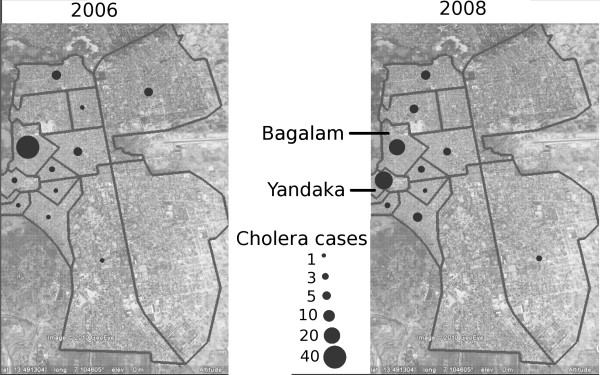
Age distribution of the cholera cases reported in the region of Maradi from 2006 to 2009 and age distribution of the inhabitants of the region of Maradi in 2001.

**Figure 5:**
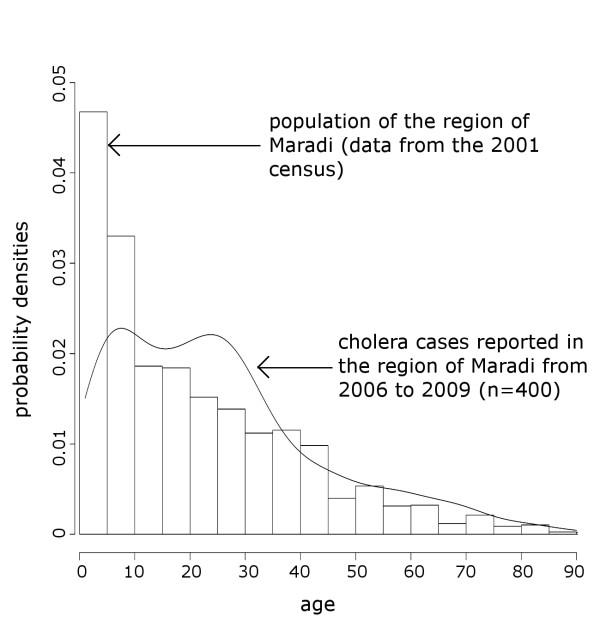
Distribution of cholera cases by neighborhood in the city of Maradi.

Surveillance system data from 2006–2009 showed the following epidemiologic indicators. Four hundred and three cholera cases were reported in the region between 2006 and 2009, among which 18 (4.47%) deaths were reported. No cases were reported in 2009. All epidemics occurred during the rainy season (Figure 2). Compared to the overall incidence rate of 0.05 per 1000 in the region for 2006 through 2009, the Maradi city incidence rate was 0.27 per 1000 for the same period (Table 3 - Figure 3). From 2006 to 2009, the mean age of cholera cases was 25 years (Figure 4). During this period, 232 women and 170 men were reported as cholera cases (sex ratio = 0.73, sex not available for 1 case). In the city, the deprived neighborhoods of Bagalam and Yandaka, which represent 1% of the regional population, reported 21% of the cholera cases (n = 85). Incidence rates of 3 per 1000 were recorded in those neighborhoods in 2006 or 2008 (Table 4 - Figure 5).

**Table 3:** Cholera cases reported to the national surveillance system between 2006 and 2009 by administrative district

Administrative districts of the region of Maradi^*^	**2006**		**2007**		**2008**		**Total 2006-2009**^**†**^	
	**Cholera cases**	**Incidence**^**‡**^	**Cholera cases**	**Incidence**^**‡**^	**Cholera cases**	**Incidence**^**‡**^	**Cholera cases**	**Incidence**^**‡**^
Aguié	0	0.00	18	0.07	0	0.00	18	0.02
Dakoro	0	0.00	0	0.00	0	0.00	0	0.00
Guidan Roumdji	6	0.02	0	0.00	4	0.01	10	0.01
Madarounfa	130	0.46	0	0.00	1	0.00	131	0.11
City of Maradi	73	0.50	0	0.00	86	0.58	159	0.27
Mayahi	0	0.00	0	0.00	30	0.08	30	0.02
Tessaoua	0	0.00	0	0.00	55	0.16	55	0.04
Total region	209	0.09	18	0.01	176	0.08	403	0.05

^*^The city of Maradi is listed separately although it is included in the administrative district of Madarounfa.

^†^No cholera cases were reported in 2009.

^‡^Incidence rates per 1000 inhabitants.

**Table 4:** Cholera cases in the city of Maradi reported to the national surveillance system between 2006 and 2009 by neighborhood

**Neighborhoods of the city of Maradi**	**2006**		**2008**		Total 2006-2009^*^	
	Cholera cases	Incidence^†^	Cholera cases	Incidence^†^	Cholera cases	Incidence^†^
Ali Dan Sofo	0	0.00	2	0.25	2	0.06
Bagalam	40	3.08	19	1.46	59	1.14
Bourja	1	0.09	0	0.00	1	0.02
Bouzou Dan Zamba	1	0.10	0	0.00	1	0.03
Dan Goulbi	1	0.21	1	0.21	2	0.11
Limantchi^‡^	1		2		3	
Makoyo	2	0.29	3	0.43	5	0.18
Maradoua	1	0.06	6	0.37	7	0.11
Mazadou Djika	1	0.27	0	0.00	1	0.07
Nouveau carré^‡^	0		5		5	
Sabon Gari	5	0.24	4	0.19	9	0.11
Soura Bildi	6	0.36	6	0.36	12	0.18
Yandaka	2	0.25	24	2.99	26	0.81
Zaria	5	0.19	0	0.00	5	0.05
Neighborhood not documented	7		14		21	

^*^No cholera cases were reported in 2007 and 2009.

^†^Incidence rates per 1000 inhabitants.

^‡^Population figures not available.

The Maradi region met the WHO criteria for endemic cholera, while Bagalam and Yandaka neighborhoods were identified as consistent high-risk areas for cholera within the city of Maradi and met the criteria for cholera vaccination according to the WHO recommendations.

## Discussion

We evaluated the cholera surveillance system of the region of Maradi and found the system sufficient to appropriately identify endemic and high risk areas for potential cholera vaccination.

Overall, the evaluation of the surveillance system suggests that the system is reasonably sensitive and that collected data are representative of the distribution of cholera cases in the region. Initial cholera cases were missed, but most were subsequently identified and recorded. Furthermore, the systematic realization of stool cultures in suspected cholera clusters and the high transmissibility of the disease made it unlikely that an epidemic of cholera would evade the surveillance system.

Few missing values were found. This may be due to the regional centralization of the surveillance system during cholera epidemics where data are checked daily at the regional level. This increased communication and feedback loop between local and regional levels helps to improve data quality. Interviews with surveillance system officers also asserted that seeking health care in case of diarrhea during a cholera epidemic was common. Thus, for people older than 5 years of age, and especially in areas with health care facilities, non-reported severe cholera cases should have been rare. The data issued from Maradi’s surveillance system could be improved, but the quality sufficed to identify high-risk areas with confidence.

Several limitations should be kept in mind when interpreting the results of the surveillance system assessment. First, the evaluation was retrospective and limited to a four year observation period during which only two had a large number of reported cases. Second, due to logistical constraints, information about cholera surveillance outside Maradi city was filled by regional heads only, while within the city all the heads of health facilities were also interviewed. In all cases, however, it was possible to contrast findings from interviews with surveillance system reports. Third, as there was no alternative surveillance system for comparison, the evaluation was based solely on a retrospective review of MoH records, reports and personal interviews. Consequently sensitivity was assessed qualitatively. Fourth, initial clinically suspected cases were laboratory confirmed, but as testing ceased after the epidemic was confirmed, a specific study would be necessary to fully evaluate the system’s PPV. Lastly, the surveillance system was designed to capture cases 5 years of age and older. While this follows WHO surveillance recommendations, it does necessarily result in an underestimation of cases in this age-group.

As an estimated 40% of the population resides more than 5 km from a health facility [14], under-reporting from these more distal areas could lead to inaccurate epidemiological estimations. In fact, sensitivity at the onset of epidemics appears to have been lower in rural areas, especially in remote villages and health centres. Due to cholera’s severity and high transmissibility, however, and supported by the results of our evaluation, it appears that most originally missed cases were retrospectively recorded by health authorities. Conversely, an overestimation of cholera cases may have occurred, particularly in urban areas due to over-attribution of non-cholera diarrheas. The clustering of cholera cases in Maradi, in addition to most neighborhoods reporting no cases suggests that over-reporting did not play a major role either.

An additional limitation applies to the calculation of incidence rates. Population data from the 2001 census would likely underestimate the population figures during the period 2006–2009, resulting in an overestimation of incidence rates in the study period. Cholera caseload could, however, as noted in Bangladesh be several-fold higher than the figure presenting at hospitals [15]. Furthermore, the system does not routinely report cases under 5 years of age, which in some Asian and African settings present with higher attack rates and are more prone to be hospitalized during cholera epidemics [16,17]. Consequently it is more likely that cholera incidence rates are higher than presented here, despite the population underestimation.

Our evaluation suggests that the cholera surveillance system in place during the reviewed period is reasonably sensitive and could be used for detection of areas at risk for cholera epidemics. WHO criteria to implement a vaccination campaign [8] were met in the neighborhoods of Bagalam and Yandaka in the city of Maradi. The city of Maradi is the major transport trade and agricultural hub of the region. These neighborhoods are also among the poorest of the city, over-crowded and are susceptible to flooding. These factors may explain why the city took a major part in the spread of cholera epidemics, particularly in the overcrowded, poor sanitation neighborhoods of Bagalam and Yandaka. Following this evaluation and identification, these two neighborhoods are being considered for a cholera vaccination campaign. Of the two currently available prequalified oral cholera vaccines, Shanchol does not require a buffer or water for administration, is less expensive and potentially promising for use in contexts with limited sanitation infrastructures [18]. In overcrowded, poor sanitation neighborhoods in the city of Beira, Mozambique, Jeuland *et al* estimated that a vaccination campaign targeting the whole 1–14 year-old population would be very cost-effective [19], despite the logistical difficulties to its implementation. The implementation of a similar long-term oral cholera vaccination campaign in the identified neighborhoods of Bagalam and Yandaka may present similar results, until associated water and sanitation infrastructure are improved.

## Conclusion

By evaluating the surveillance system and subsequently the surveillance data, the Maradi region could be considered endemic for cholera. Two high risk neighborhoods in the regional capital were identified as candidates for preventive cholera vaccination. This case study shows that evaluation of surveillance systems and the use of its data, when reliable, can be an efficient approach for the identification of high-risk areas for cholera in low- and middle-income settings before considering cholera vaccination campaigns.

## Abbreviations

WHO, World Health Organization; MOH, Ministry of Health; PPV, Positive Predictive Value.

## Competing interests

No author reports a competing interest.

## Authors’ contributions

JG collected and analyzed the data and drafted the manuscript. All authors contributed to the design and interpretation of the data and to revising the manuscript. All authors read and approved the final manuscript.
